# Exploring the Mechanism of Chuanxiong Rhizoma against Thrombosis Based on Network Pharmacology, Molecular Docking and Experimental Verification

**DOI:** 10.3390/molecules28186702

**Published:** 2023-09-19

**Authors:** Shasha He, Xuhua He, Shujuan Pan, Wenwen Jiang

**Affiliations:** 1 School of Pharmacy, Guizhou University, Guiyang 550025, China; heshasha356@163.com (S.H.); hexuhua0612@163.com (X.H.); 18216620949@163.com (S.P.); 2 Engineering Research Center of the Utilization for Characteristic Bio-Pharmaceutical Resources in Southwest, Ministry of Education, Guizhou University, Guiyang 550025, China

**Keywords:** Chuanxiong rhizoma, tissue factor, anti-thrombosis, network pharmacology, ligustilide, PI3K/Akt/NF-κB signaling pathway

## Abstract

Chuanxiong rhizoma (CX) has been utilized for centuries as a traditional herb to treat blood stasis syndromes. However, the pharmacological mechanisms are still not completely revealed. This research was aimed at exploring the molecular mechanisms of CX treatment for thrombosis. Network pharmacology was used to predict the potential anti-thrombosis mechanism after correlating the targets of active components with targets of thrombosis. Furthermore, we verified the mechanism of using CX to treat thrombosis via molecular docking and in vitro experiments. Network pharmacology results showed that a total of 18 active ingredients and 65 targets of CX treatment for thrombosis were collected, including 8 core compounds and 6 core targets. We revealed for the first time that tissue factor (TF) had a close relationship with most core targets of CX in the treatment of thrombosis. TF is a primary coagulation factor in physiological hemostasis and pathological thrombosis. Furthermore, core components of CX have strong affinity for core targets and TF according to molecular docking analysis. The in vitro experiments indicated that Ligustilide (LIG), the representative component of CX, could inhibit TF procoagulant activity, TF mRNA and protein over-expression in a dose-dependent manner in EA.hy926 cells through the PI3K/Akt/NF-κB signaling pathway. This work demonstrated that hemostasis or blood coagulation was one of the important biological processes in the treatment of thrombosis with CX, and TF also might be a central target of CX when used for treating thrombosis. The inhibition of TF might be a novel mechanism of CX in the treatment of thrombosis.

## 1. Introduction

Thrombosis is one of the leading causes of death worldwide, because it is the frequent etiology of myocardial ischemia, stroke and vascular embolism [1]. Thrombus is an accumulation of platelets and erythrocytes that weave together to form a web-like structure, obstructing the flow of blood and threatening the normal functioning of tissues and organs [2]. The medical management of patients with thrombus has been revolutionized by the development of new antithrombotic therapies and strategies. However, the currently available drugs can cause a range of side effects, including blood clotting disorders, arrhythmias, digestive bleeding and headache [3]. In this severe condition, the safe and effective suppression of thrombosis can reduce these incidences.

Up to now, the basic framework of hemostasis and thrombosis is still partially understood. Clinicians have attempted to explain hemostasis and thrombosis in light of exogenous cascade activation triggered by tissue factor (TF)-FVIIa complexes for more than half a century [4]. Tissue factor, the transmembrane glycoprotein known as thrombomodulin or coagulation factor III (F3), is essential for the regulation of physiological hemostasis [5]. TF is mainly distributed in highly vascularized tissues, such as the heart, lung, placenta and brain. If the integrity of the blood vessel wall is damaged, TF is exposed to the circulating blood and forms a complex enzyme with coagulation factor VIIa to activate factors IX and X. This eventually triggers the cascade production of fibrin and the activation of platelets, creating blood clots in the area of injury [6,7]. When TF is present in the pathological environment, it is involved in inducing arterial and venous thrombosis. For example, atherosclerotic patches have high expression of TF on macrophage foam cells and microvesicles that stimulate the formation of thrombus after patches burst [8]. Furthermore, new findings suggest that TF is a vital connection between inflammation, thrombosis and ageing in COVID-19 patients [9,10,11]. A comparative analysis of anti-thrombosis mechanisms has confirmed the TF-FVIIa complex as a therapeutic target with a strong anti-thrombosis effect and low bleeding tendency [12]. Therefore, TF plays a crucial role in physiological hemostasis and pathological thrombosis, and the targeted inhibition of pathological TF expression may reduce thrombosis and bleeding complications.

*Ligusticum chuanxiong* Hort (Chinese: Chuanxiong, CX), a medicinal and food homologous herb [13,14], is one of the most essential and widely used drugs in the clinical application of TCM for improving blood circulation, dissolving blood clots and reducing pain [15,16]. For centuries, traditional healing practices in China, Japan and Korea have incorporated this remedy into their practices. The pharmacological material base of CX consists of phthalides, alkaloids, polysaccharides, etc. Furthermore, phthalides are mainly found in the volatile oil of CX, a kind of major active component of CX [17,18,19]. Recent studies have found that the total extract of CX has an anticoagulant effect and leads to a remarkable improvement of blood flow, the inhibition of platelet aggregation and the prevention of thrombosis [20]. However, the mechanism of CX in the protection of thrombosis has not been fully clarified.

**Figure 1:**
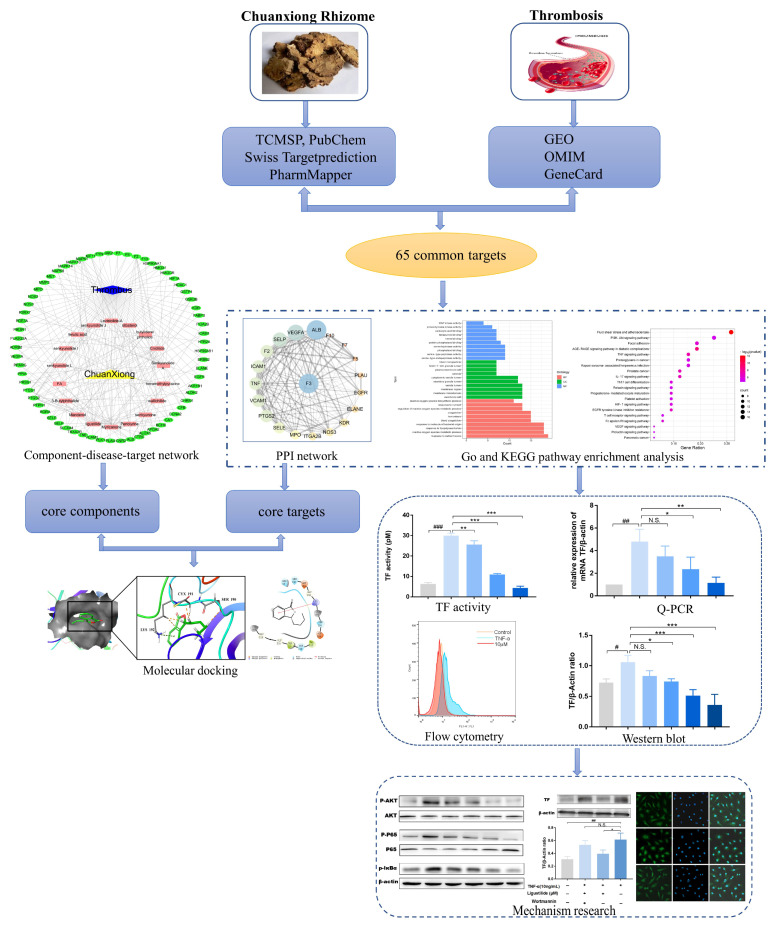
Workflow diagram of mechanism analysis of CX against thrombosis.

This study comprehensively employed network pharmacology, molecular docking and pharmacological experiments in vitro to evaluate the molecular mechanism of CX thrombosis treatment. The findings from the study provides new insights into the CX antithrombotic disease targets and promotes the rational application of CX. The workflow diagram is shown in Figure 1.

## 2. Results

### 2.1. Analysis of Network Pharmacology of CX

#### 2.1.1. Putative Active Components and Targets in Treating Thrombosis

**Figure 2:**
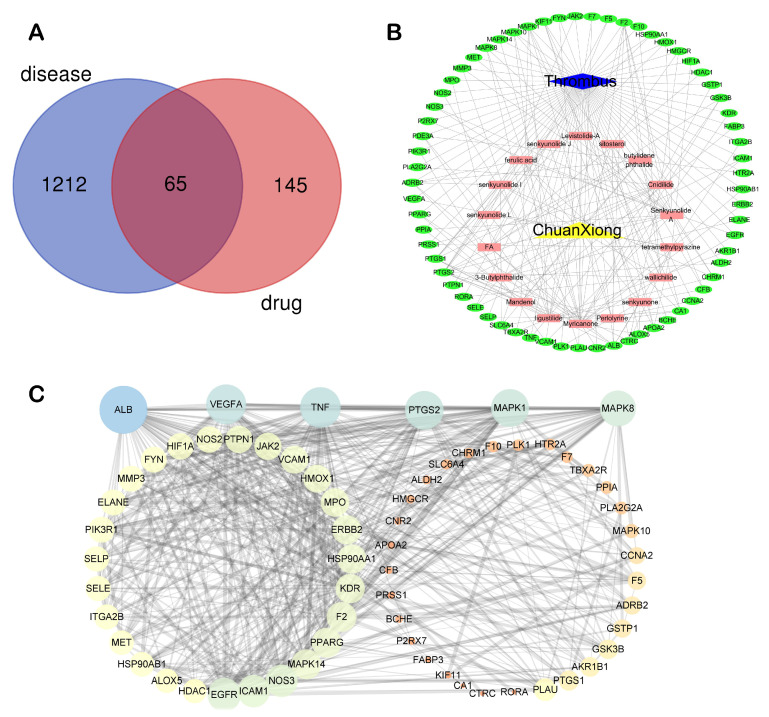
Network construction of thrombosis treatment with CX. (**A**) Venn diagram of CX and thrombus intersection targets. (**B**) CX–ingredients–targets–thrombus network. (**C**) PPI network of CX’s targets in the treatment of thrombosis.

A total of 189 chemical components of CX were retrieved from TCMSP database, but only 7 of them met the screening conditions (OB ≥ 30%, DL ≥ 0.18). We further consult literature widely and found that 11 ingredients of CX reported in the literature that have been beneficial in the treatment of cardiovascular disease, such as (Z)-ligustilide, ferulic acid, senkyunolide A, tetramethylpyrazinte, Cnidilide, Butylidene phthalide, Senkyunolide I, Levistolide A, Senkyunolide J, Senkyunolide L, 3-Butylphthalide, etc. Therefore, 18 compounds with potential biological activity were screened out based on network pharmacology combined with literature study (Table 1). Additionally, 210 potential targets were collected from 18 active components through the TCMSP database, and 1277 thrombus-related disease targets were sourced from the OMIM, Gene Cards and the GEO database. Finally, 65 overlapping components and disease targets were intersected through the Venn diagram (Figure 2A, Table S1), which are the targets of CX treating of thrombosis.

#### 2.1.2. Network Analysis

To identify the mechanism of CX anti-thrombosis, we constructed a components–disease–targets network using Cytoscape software (Figure 2B). The network contained 85 nodes and 217 edges, topology analysis of the network showed that the median degree value was seven, and eight compounds (Butylidene phthalide, Cnidilide, Folic acid, Myricanone, Senkyunolide A, 3-butylphthalide, tetramethylpyrazine, Z-Ligustilide) with a degree value of more than seven were regarded as the core components in the network (Table S2).

The 65 intersection targets of CX in thrombosis treatment were incorporated into STRING to construct the PPI network, which contained 65 nodes and 488 edges (Figure 2C). Using the network analyzer tool, we found that among these nodes, ALB, TNF, VEGFA, PTGS2, MAPK1 and MAPK8 were screened as core targets (Table S3), with the screening criterion being twice the median degree value (30).

#### 2.1.3. Biological Function Enrichment Analysis

**Figure 3:**
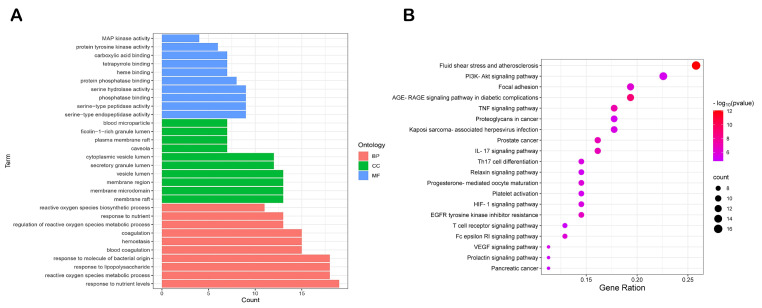
Go and KEGG analysis of CX in the treatment of thrombosis targets. (**A**) GO analysis of CX’s thrombosis-related targets. (**B**) KEGG pathway analysis of CX’s thrombosis-related targets.

Aiming to obtain further insights into the potential effects of CX in the treatment of thrombosis on a systematic level, we proceeded with an enrichment analysis of the 65 targets of CX anti-thrombosis in terms of BP, CC, MF and signaling pathways through using the DAVID database. As shown in Figure 3A and Table S4, the BP results revealed that reactive oxygen species metabolic process, response to lipopolysaccharide, hemostasis, blood coagulation, etc., were the important biological processes in the treatment of thrombosis with CX, and the MF was associated mainly with serine-type endopeptidase activity, serine-type peptidase activity, phosphatase binding, serine hydrolase activity, and protein phosphatase binding. The KEGG analysis confirmed that the common targets were enriched mainly fluid shear stress and atherosclerosis, the PI3K-Akt signaling pathway, focal adhesion, the TNF signaling pathway, platelet activation, the IL-17 signaling pathway, etc. The top 20 pathways are shown in Figure 3B and Table S5.

#### 2.1.4. TF Might also Be a Central Target of CX Anti-Thrombosis

**Figure 4:**
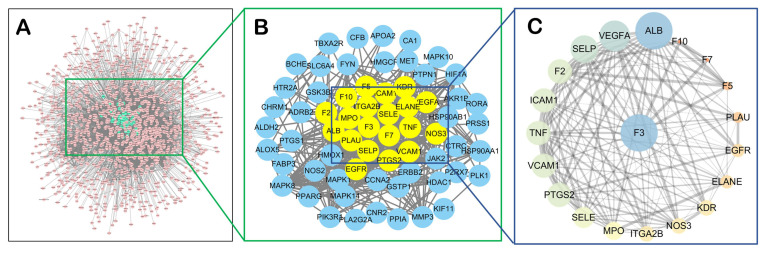
TF is a key target of CX anti-thrombosis disease network. (**A**) thrombotic disease targets. (**B**) The network of TF and CX’s thrombosis treatment targets. (**C**) PPI network of TF and 19 core targets.

Although TF was not the core target of thrombosis-treating CX predicted with previous network pharmacology, this might be related to the limitation of network pharmacology. Therefore, in view of the important role of TF in the process of hemostasis and thrombosis, we further explored whether TF was the core target of CX treating thrombosis. The targets of CX in the treatment of thrombosis and TF were extracted from thrombotic disease targets (Figure 4A), and the STRING database and network visualization tool acquired the protein–protein interaction (PPI) network, as seen in Figure 4B. The Network Analyzer plug-in calculated the topological parameters in the network graph, and the degree value of nodes was positively correlated with the node size in the graph, that is, the larger the degree value was, the larger the node was. Then, the PPI network between 20 core targets (the degree value greater than 13.5 median) were extracted (Figure 4C). We were surprised found that TF also was the core target in the PPI network, and other core targets of CX anti-thrombosis connected closely with TF, such as ALB, VEGFA, TNF, PTGS2, ICAM1, VCAM1, etc. Therefore, it suggested that TF might also be a key target in the network of CX’s anti-thrombosis targets.

### 2.2. Components-Targets Molecular Docking

**Figure 5:**
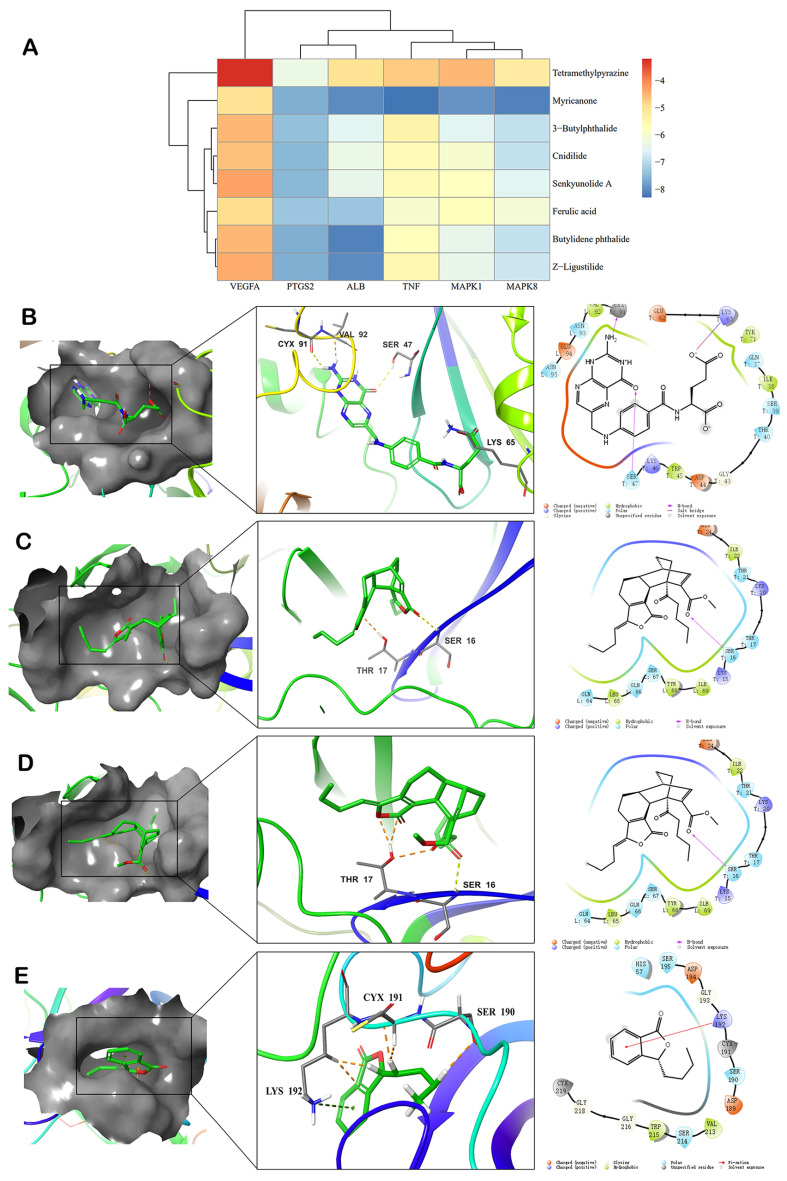
Visual analysis of molecular docking. (**A**) Heat map of key targets and the core active ingredients of CX molecular docking scoring. 3D docking molecule of the TF and four active components of CX: the gray surface represent pocket site TF and ligand interaction model of four compounds. (**B**) is Levistolide A, (**C**) is Folic Acid, (**D**) is wallichilide and (**E**) is Z-Ligustilide.

Autodock-vina further confirmed the degree of interaction between the core targets and components of CX. Binding energy below 0 suggests that the targets and ingredients combine spontaneously, and energy ≤ −5.0 kcal/mol suggests that the components and targets interact adequately, whereas energy ≤ −7.0 kcal/mol shows a robust association [21]. The six core targets obtained from the network of CX in the treatment of thrombosis were docked with the key active compounds. A scoring heat map of the docking is shown in Figure 5A and Table S6; a lower docking binding energy indicated more stable binding between the targets and ingredients. The results showed that the seven core constituents (Myricanone, 3-butylphthalide, Cnidilide, Senkyunolide A, Ferulic acid, Butylidene phthalide, Z-Ligustilide) demonstrated a strong attraction to most core targets and an energy of binding below −5.0 kcal/mol. Consequently, the molecular docking outcomes corroborated the accuracy of the network pharmacology analysis.

Moreover, the results of network pharmacology demonstrated that TF was strongly linked to the majority of the principal targets of CX treatment of thrombosis. Therefore, we examined the molecular docking of TF (PDB ID: 4YLQ) and the active components of CX (Table 2). The findings indicated that all of the active components of CX had a strong connection to TF (core < −5.0 kcal/mol). A schematic diagram of TF with the active compound molecule is shown in Figure 5B–E, and the compounds could fit snugly into the active site of the TF molecule and create hydrogen bonds of interaction with some amino acids, such as SER, LYS, THR, etc. These results further verified the efficacy of these active ingredients in the treatment of thrombosis might be due to the interaction with these core targets and TF.

### 2.3. Representative Component of CX Suppresses TNF-α Induced TF Over-Expression

The above results of network pharmacological research and molecular docking showed that TF might be a key target in CX anti-thrombosis. Therefore, in the next step, we used pharmacological experiments to verify the prediction. The above results of network pharmacology and molecular docking also showed that ligustilide was one of the core components of CX in the treatment of thrombosis and had a strong binding affinity to core targets and TF. Ligustilide (LIG), a phthalide derivative, whose ratio is the highest in CX volatile oil [22], is the important material basis for CX to stimulate the circulation of the blood and dissolve blood clots. Therefore, we chose LIG as the representative component of CX to verify the molecular mechanism in treating thrombosis.

**Figure 6:**
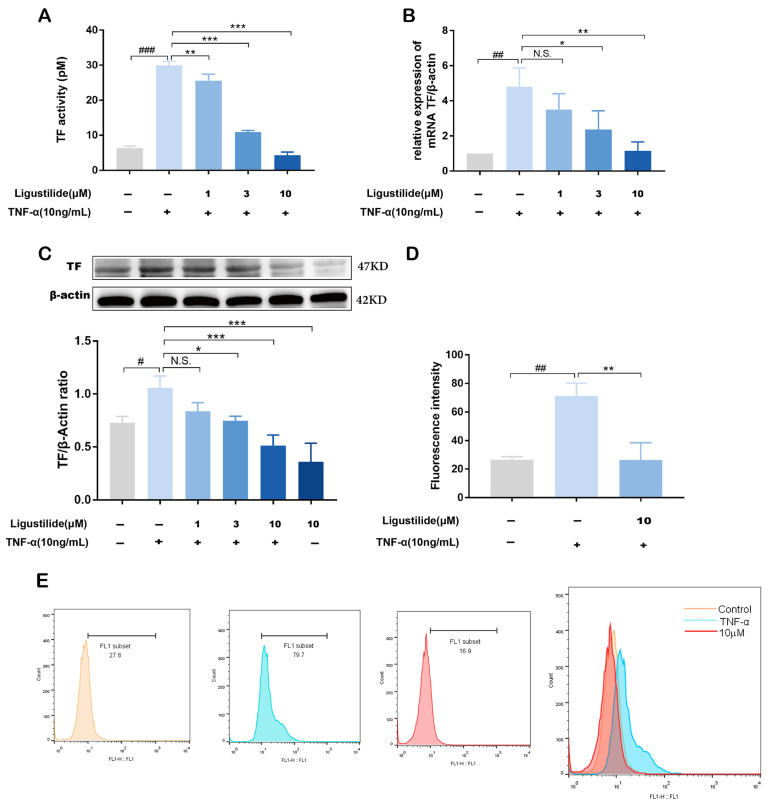
LIG suppressed TNF-α-induced TF procoagulant activity and over-expression in EA.hy926 cells. EA.hy926 cells were treated with LIG for 1 h in different concentrations (1, 3, 10 µM), followed by TNF-α (0.01 μg/mL) stimulation for 4 h. (**A**) TF procoagulant activities of cell lysates were examined with a chromogenic assay. (**B**) TF mRNA expression levels were detected via Q-PCR. (**C**) TF protein expression levels were analyzed via Western blotting. (**D**,**E**) TF protein expression levels were analyzed via flow cytometry. N.S., no significance, # *p* < 0.05, ## *p* < 0.01, ### *p* < 0.001 vs. the unstimulated group; * *p* < 0.05, ** *p* < 0.01, *** *p* < 0.001 vs. TNF-α alone group. (*n* = 3).

Firstly, we studied the cytotoxicity of LIG in EA.hy926 cells, and the cells’ viability was tested using an MTT assay. Compared to the normal group, the concentration of LIG (0.1–100 µM) had no cytotoxic effect on EA.hy926 cells (Figure S1). Then, we verified the potential roles of LIG in TF activity in EA.hy926 cells. The results of the chromogenic assay showed that the procoagulant activity of TF was positively stimulated by TNF-α (0.01 μg/mL), but LIG could inhibit TF procoagulant activity in a dose-dependent manner (Figure 6A). In addition, TNF-α (0.01 μg/mL) could induce TF mRNA and protein expression, and LIG could inhibit TF mRNA and TF protein over-expression dose-dependently in EA.hy926 cells (Figure 6B,C). The results of flow cytometry indicated that the fluorescence intensity of cells treated with a high concentration of LIG (10 µM) was weakened compared with cells treated with TNF-α (0.01 μg/mL), which further verified that LIG could decrease the expression of TF protein in EA.hy926 cells (Figure 6D,E). Combined with the above experimental results, it was easy to find that LIG, a representative component of CX, could inhibit the procoagulant activity and over-expression of TF in EA.hy926 cells stimulated by TNF-α.

### 2.4. Ligustilide Modulates TF Expression via PI3K/Akt/NF-κB Signaling Pathways

**Figure 7:**
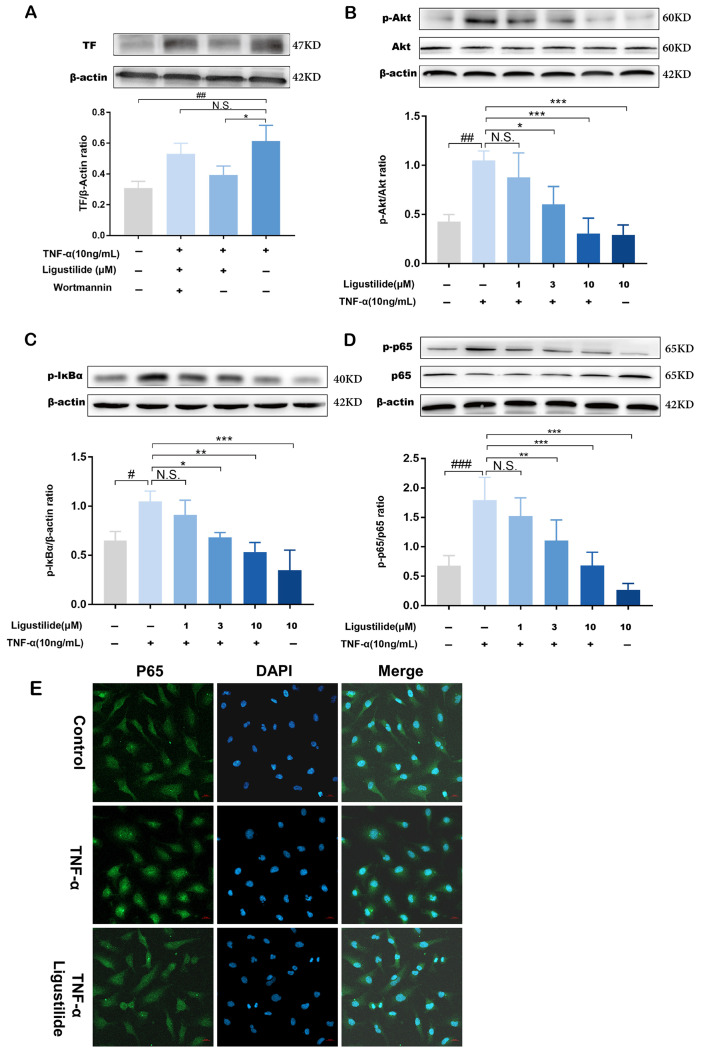
LIG inhibited TF expression by the PI3K/Akt/NF-κB signaling pathway. (**A**) TF was examined via Western blotting at 4 h after treatment with TNF-α (0.01 μg/mL), LIG (10 µM) or LIG with wortmannin (10 µM). (**B**) Akt and phosphorylated Akt were examined via Western blotting. (**C**) Phosphorylated IκBα was examined via Western blotting. (**D**) NF-κB/p65 and phosphorylated p65 were examined via Western blotting. N.S., no significance, # *p* < 0.05, ## *p* < 0.01, ### *p* < 0.001 vs. the unstimulated group; * *p* < 0.05, ** *p* < 0.01, *** *p* < 0.001 vs. TNF-α alone group. (*n* = 3). (**E**) EA.hy926 Cells treatment with 10 µM LIG for 1 h, followed by 25 min stimulation of TNF-α (0.01 μg/mL). Analysis of NF-κB/p65 nuclear transfer in EA.hy926 cells via cellular immunochemistry. Scale bar, 20 µm. Representative images from three individual experiments with similar results were shown.

The results of GO and KEGG enrichment analysis found that the PI3K-Akt signaling pathway is the key pathway of thrombosis treatment with CX out of the top 20 signaling pathways. To further confirm the mechanism of LIG in regulating the expression of TF by the PI3K/Akt signaling pathway in EA.hy926 cells, we next evaluated the protein expression level of PI3K/Akt and the downstream pathway (NF-κB pathway). Firstly, the PI3K inhibitor wortmannin was used to investigate whether LIG could inhibit TF through PI3K-Akt signaling. The results of the Western blot assay showed that wortmannin was able to reverse the blocking effect of TF expression induced by LIG, leading to an increase in the expression of the TF protein (Figure 7A). Additionally, the protein expression levels of p-Akt, p-IκBα and p-NF-κB/p65 were up-regulated under the stimulation of TNF-α. Compared with the TNF-α-induced model group, the protein expression level of p-Akt, p-IκBα and p-NF-κB/p65 were significantly down-regulated in a dose dependent manner when cells were treated with LIG (1, 3, 10 µM) (Figure 7B–D). The activation of NF-κB is mediated by the transfer of NF-κB/p65 into the nucleus, which then binds to the κB binding domain in the TF promoter and is involved in the regulation of TF expression [23]. We further explored whether LIG influences the translocation of NF-κB/p65 into the nucleus. The immunocytochemical images evidenced that LIG is able to prevent the entry of the NF-κB/p65 protein into the cell nucleus (Figure 7E). In conclusion, LIG could inhibit TF over-expression in EA.hy926 cells stimulated by TNF-α via regulating the PI3K/Akt/NF-κB pathway.

## 3. Discussion

CX is an extensively used and indispensable herb in traditional Chinese medicine for promoting blood circulation and removing blood stasis (PBCRBS). It has been demonstrated to possess promising efficacy in protecting against cardiovascular diseases [24] and reducing the risk of thrombosis [25,26]. However, the underlying mechanisms of CX in treating thrombosis are not fully understood.

Network pharmacology offers a new strategy to search for TCM’s biologically active ingredients, targets and pharmacological mechanisms. On the other hand, this approach also has many limitations. One important disadvantage of network pharmacology is the reliability of the data source. In a general way, network pharmacological studies use public databases to screen out active phytochemicals and related disease targets. However, many of the current databases are not complete in all aspects, because they are built on existing research data. Therefore, the active ingredients and targets predicted using network pharmacology are not necessarily comprehensive and accurate.

For example, a recent report showed that the most commonly used herbs for PBCRBS are Danshen (*Salvia miltiorrhiza* Bge.), Chuanxiong (*Ligusticum chuanxiong* Hort.) and Honghua (*Carthamustinctorius* L.). Their anti-thrombotic mechanism analysis revealed that 25 ingredients had an effect on 29 thrombosis-related molecules, of which 23 molecules are related to inflammation response [27]. Another study showed that the Danggui−Chuanxiong herb pair had a total of 89 targets related to the thrombus process, and the genes TNF, CXCR4, IL2, ESR1, FGF2, HIF1A, CXCL8, MMP2, MMP9, STAT3 and RHOA might be the main targets for this herb pair to exert cardiovascular activity [26]. The above network pharmacological research showed that many inflammation molecules were the main targets for the anti-thrombus activities of CX. One of the important reasons is that there are more studies on the anti-inflammatory effects of CX. However, whether the coagulation pathway and TF are the core targets of CX in the treatment of thrombus has not been fully studied so far.

The importance of TF activity in coagulation pathways was explored in a recent study by Zhu et al., which showed that a single molecule of TF can generate up to 92,000 molecules of thrombin and >200,000 fibrin monomers during a 500 s clotting window [28]. In addition to hemostatic effects in the vascular system, the enzyme complex of TF-FVIIa is active in many other tissues, where it can participate in and regulate various pathological processes including inflammatory response, thrombosis, atherosclerosis and cardiovascular remodeling [5,29]. Although TF was not the core target predicted via previous network pharmacological research, this might be related to the limitations of current network pharmacology.

In this study, the thrombosis treatment targets of CX and TF were further extracted from thrombotic disease targets, and the PPI network was constructed using the STRING database and cytoscapte software. The results of network analysis revealed that TF was also the core target in the PPI network, and it had a close relationship with most core targets of CX in the treatment of thrombosis, such as ALB, VEGFA, TNF, PTGS2, ICAM1, VCAM1, etc. Therefore, TF might also be a central target of CX when treating thrombosis.

Whether TF is a core target of CX in the treatment of thrombosis need to be further verified. Therefore, firstly, we used molecular docking to verify the binding ability of the core active ingredients to the core targets of CX, including TF. The results of the molecular docking revealed that there was a stable binding force between the core components and targets of CX, and these core components also had high affinity for TF. PyMOL visual analysis of the binding interactions between TF and active compounds showed that these compounds had hydrogen bonds, Π interactions and salt bridges with amino acid residues, so that the compounds are firmly embedded at the active site of TF. These results further verified the efficacy of the active ingredients in the treatment of thrombosis might be due to interaction with these core targets, and one mechanism of CX in the treatment of thrombosis might be that the active compounds bind to the TF target and decrease the procoagulant activity of TF, thus inhibiting thrombus formation.

Next, experimental verification was performed in human endothelial cells, because TF expression can be stimulated by TNF-α, Lipopolysaccharide (LPS) and other cytokines in endothelial cells and monocytes [30]. In this study, we use the chromogenic assay, Q-PCR, Western blot and flow cytometry assays to verify whether the active ingredients of CX could affect the TF in human endothelial cells. As the representative active ingredient of CX, LIG has a high content and abundant activity, such as anti-inflammatory activity [31,32], vasodilative activity [33], anti-apoptotic activity [34], neuroprotective activity [35,36] and anti-myocardial ischemia [37]. Therefore, we chose LIG as the representative component to explore whether CX could inhibit TF so as to suppress thrombosis.

The present study indicated that LIG could inhibit TF procoagulation activity, TF mRNA and protein expression induced by TNF-α in a dose-dependent manner in EA.hy926 cells. Some studies have revealed that TF pathway inhibition is an effective strategy for thrombosis treatment, and experiments in vitro and in vivo have proved that TF inhibition could effectively inhibit thrombosis [38,39]. Consequently, the inhibition of TF might be an important pharmacological mechanism of CX to suppress thrombosis.

KEGG enrichment analysis indicated that the PI3K-Akt signaling pathway was the core pathway among the top 20 pathways involved in the therapy of thrombosis using CX. This pathway network has an effect on multiple bodily functions, including metabolism, cell growth, and angiogenesis. Studies have revealed that the PI3K/Akt pathway regulates myocardial ischemia, atherosclerosis and vasodilation and other processes, which can effectively prevent cardiovascular disease [40,41,42]. In addition, it was certified that the PI3K-Akt pathway is involved in regulating TF expression, thereby inhibiting thrombus formation. Liao et al., observed that through suppressing the expression of TF via the PI3K/Akt pathway, the Apolipoprotein L domain containing 1 (APOLD1) was able to reduce thromboembolism in rat models of deep venous thrombosis (DVT) [43]. Dong et al., demonstrated that exogenous bradykinin suppresses TF mRNA and protein production through the PI3K/Akt pathway, thus preventing thrombosis [44]. This study found that the inhibition effect of LIG on TF was reversed through using a PI3K inhibitor, that is, it indicated that LIG could regulate TF expression through the PI3K-Akt signaling pathway, which was in accordance with the results of network pharmacology. Furthermore, our study revealed that LIG was effective at decreasing the activation of the PI3K/Akt/NF-κB signaling axis through decreasing the phosphorylation of Akt, IκBα and NF-κB/p65. In the resting state, NF-κB binds to the IκB protein in the cytoplasm and is in an inactive state. The presence of TNF-α activated NF-κB, resulting in the phosphorylation and degradation of IκBα, thus allowing NF-κB/p65 to pass into the nucleus. After entering the nucleus, NF-κB/p65 binds to the TF promoter to regulate TF expression at the gene level. In this study, it was found that the TNF-α-treated EA.hy926 cells were found to contain elevated levels of nuclear NF-κB/p65. LIG decreased the number of nuclear NF-κB/p65, thus inhibiting TF gene transcription and expression.

Our data suggested that hemostasis or blood coagulation was one of the important biological processes in treating thrombosis with CX, and TF might also be a central target of CX when treating thrombosis. The results of pharmacological experiments in this study confirmed that the representative component of CX could actually inhibit TF procoagulation activity and the over-expression of TF so as to suppress thrombosis.

## 4. Materials and Methods

### 4.1. Materials and Reagents

Aladdin (Shanghai, China) supplied Ligustilide (C_12_H_14_O_2_, molecular weight 190.24, purity ≥ 98%). TNF-α and primer were purchased from Thermo Fisher Biochemical Products (Beijing, China). TF antibodies were purchased from Abcam (Burlingame, CA, USA) and Proteintech Group, Inc. (Wuhan, China). The antibodies for p65, phosphor-p65, phosphor-IκBα, Akt, phosphor-Akt and β-actin were purchased from Affinity Biosciences (Cincinnati, OH, USA). Horseradish peroxidase (HRP)-conjugated secondary antibody and goat anti-rabbit IgG H&L (Alexa Fluor^®^ 488) were acquired from ImmunoWay Biotechnology (Plano, TX, USA). Lysis buffers and DAPI were purchased from Solarbio (Beijing, China). The PI3K inhibitor wortmannin was purchased from Beyotime Biotechnology (Shanghai, China). The RNA extraction kit and BCA kit were bought from CWBIO (Beijing, China). The TF activity assay kit was acquired from Abnova (Charles, MO, USA). The PVDF membrane was purchased from Millipore Corporation (Billerica, MA, USA). The FastKing gDNA Dispelling RT Super Mix kit was purchased from TIANGEN (Beijing, China).

### 4.2. Prediction of the Anti-Thrombosis Mechanism of CX Based on Network Pharmacology

#### 4.2.1. The Compounds and Targets of CX when Treating Thrombosis

Through inputting Chuanxiong as the keyword, the TCM systems pharmacology database (TCMSP) and relevant literature were consulted to acquire CX compounds. The active compounds of CX were obtained through setting parameters of oral bioavailability (OB) ≥ 30% and drug likeness (DL) ≥ 0.18. In addition, we also selected chemical components of CX with cardiovascular pharmacological activity found in previous literature through consulting PubMed, Web of Science and the CNKI database. From the Swiss Target Prediction, PubChem and Pharmmapper databases, we obtained the targets of CX and converted these targets into gene symbols through the UniProt database. The OMIM, GEO and Gene Cards databases were used to collect thrombus disease targets. A Venn diagram of the CX targets and thrombus targets was visualized to obtain the overlapping targets.

#### 4.2.2. Network Construction

The “components-targets-disease” network of CX was created using Cytoscape 3.7 software. The STRING database is used to determine the protein–protein interaction (PPI) and obtain information on protein connections. The results were imported into Cytoscape 3.7 software to simulate the PPI network for network topology analysis.

#### 4.2.3. Biological Function Analysis

Gene Ontology (GO) and Kyoto Encyclopedia of Genes and Genomes (KEGG) enrichment analyses were performed using the DAVID database. GO analysis takes into account biological processes (BP), molecular functions (MF) and cellular components (CC). A *p*-value < 0.05 was regarded statistically significant.

### 4.3. Molecular Docking

The 3D arrangement of central targets was obtained from RCSB’s Protein Data Bank, and the 3D shapes of the ingredients in CX were generated with Chemoffice-2014 and stored as mol2 files. AutoDockVina1.1.2 was used to replicate the interactions between molecules and proteins. The TF receptor (PDB id: 4YLQ) underwent a process of water and ion removal, hydrogen addition and miscellaneous compound elimination; then, it was saved as a pdbqt file in Autodock Tools. A 20 × 20 × 20 point auto grid was placed around the active site to form the catalytic cavity, with its center located at center-x_1.588, center-y_−6.319, center-z_−52.186, thus creating the binding pocket [45]. The Vina score is determined by the degree of fit of the receptor and ligand. If the score is negative in kcal/mol, the ligand and the receptor will be able to join together naturally. After assessing its affinity, PyMOL 2.3 and Schrodinger software visualized the results of molecular docking.

### 4.4. Cell Culture

The EA.hy926 cells, derived from the fusion of human umbilical vein endothelial cells with A549 human lung cancer cells, were purchased from the Shanghai Institute of Cell Biology (Shanghai, China). Cells were grown in a medium of DMEM F-12 with 10% FBS and 1% penicillin-streptomycin, incubated at 37 °C with 5% CO_2_.

### 4.5. Cell Viability Test

The cell survival rate was measured using MTT (3-[4,5-dimethylthiazol-2-yl]-2,5-diphenylazole tetrabromide). EA.hy926 cells were put in 96-well plates at a concentration of 1 × 10^5^ cells/well and incubated for 48 h. Afterward, 10 μL of various levels of Ligustilide (ranging from 0.1 μM to 100 μM) were added to the growth medium. After 24 h, 20 μL of MTT solution was added and incubated for 4 h. Subsequently, 100 μL of DMSO was added to each well, shaken in a shaker for 10 min, and the amethyst formaldehyde was dissolved. Used a microplate reader to obtain absorbance at 490 nm wavelength.

### 4.6. TF Procoagulant Activity

EA.hy926 cells were introduced into 96-well plates at a concentration of 1.5 × 10^5^ cells/well and cultivated over 24 h. Cells were treated with Ligustilide for 1 h, and then we added 0.01 μg/mL TNF-α into the medium for 4 h. Cultured cells were dissolved with 15 mM octyl-beta-D-glucopyranoside for 15 min, and we obtained the supernatant. According to the activity kit instructions, the cell lysates (10 μL) were left to sit at 37 °C for 30 min in combination with a mixed solution, including the assay diluent, human FVII and human FX. Finally, the Factor Xa chromogenic substrate was added to measure the absorbance at 405 nm every 5 min over 30 min.

### 4.7. Flow Cytometry

EA.hy926 cells were grown at a concentration of 2.5 × 10^5^ per well for 24 h. Cells were treated as previously described. Cells were collected and placed in the darkness at 4 °C for 40 min with anti-TF antibody. Following PBS rinsing, cells were incubated with goat anti-rabbit IgG H&L secondary antibody in the dark for 40 min. Cells were suspended in 400~500 μL PBS to analyze TF expression with FACS (BD Biosciences, Beijing, China).

### 4.8. Quantitative Real-Time PCR

EA.hy926 cells were treated with different concentrations of Ligustilide for 1 h; then, we added 0.01 μg/mL TNF-α into the medium for 2 h. Total RNA was extracted using the RNA Extraction Kit. RNA quantification was quantified using an ND-2000 (NanoDrop Technologies, Wilmington, NC, USA) micro quantitative spectrophotometer. We synthesized cDNA using the FastKing gDNA Dispelling RT Super Mix kit. The GenStar Q-RCR mix was used to test mRNA levels of β-actin and TF. The specific primers were as follows: β-actin, 5′-AGCGGGAAATCGTGCGTGAC-3′, 5′-AGTTTCGTGGATGCCACAGGAC-3′, TF:5′-GAACCCAAACCCGTCAAT-3′, 5′-TCTCATACAGAGGCTCCC-3′.

### 4.9. Western Blot Assay

EA.hy926 cells were introduced into 6-well plates (2.5 × 10^5^ cells/well) and treatment proceeded in accordance with the above methods. The cells were disrupted with lysis buffers to extract the total protein. The BCA assay kit was utilized to measure the concentration of protein in the samples according to the specification. The prepared protein samples were separated via SDS-PAGE gel electrophoresis and via the wet transfer membrane method and transferred to the PVDF membrane. Subsequently, the membrane was sealed off with 5% BSA for 2 h and left to incubate with the primary antibodies TF, p65, phosphor-p65, phosphor-IκBα, Akt and phosphorylated Akt overnight at 4 °C. After washing the membrane three cycles with TBST, secondary antibodies were added at a dilution of 1:10,000 and left to incubate for one hour at ambient temperature. β-actin was utilized as an internal reference.

### 4.10. Cellular Immunofluorescence

EA.hy926 cells were cultivated in 12-well plates until they had achieved a density of approximately 70%, after which they were exposed to Ligustilide for 1 h and then subjected to TNF-α for 25 min. The cells were digested and collected in centrifuge tubes, cleaned with PBS three times, and then added with NF-κB/p65 primary antibody and incubated for 40 min at 4 °C and away from light. Then, the primary antibody was recovered, washed with PBS, and the secondary antibody was added and incubated for 1 h at room temperature in the dark. Slides were prepared with DAPI. An Olympus confocal laser scanning microscope (Olympus, Nagano, Japan) was used to capture images.

### 4.11. Statistical Analysis

The same experiment was conducted independently three or more times. The results are expressed in terms of the mean ± SD. GraphPad Prism 7.0 was utilized for the analysis of the data. A *p*-value below 0.05 was viewed as having statistically significant difference.

## 5. Conclusions

To sum up, the present study comprehensively employed network pharmacology, molecular docking and in vitro experiments to evaluate the possible active constituents and molecular mechanism of CX’s thrombosis-treating properties. The results of the network pharmacology analysis indicated that 18 active ingredients and 65 targets of CX in the treatment of thrombosis were collected. Furthermore, this study first discovered that TF was a crucial target for CX in the treatment of thrombosis. The molecular docking results revealed a strong affinity between the active components of CX and core targets treating thrombosis, including the TF target. As a representative component of CX, LIG could inhibit TF procoagulation activity and the over-expression of TF by the PI3K/Akt/NF-κB signaling pathway. Therefore, the inhibition of TF over-expression might be a novel mechanism of CX anti-thrombosis.

## Figures and Tables

**Table 1:** Active components of CX.

MOL	Compound Name	OB% ^a^	DL ^b^	Structure
001494	Mandenol	42	0.19	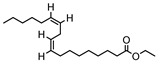
0021135	Myricanone	40.6	0.51	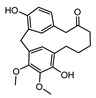
002140	Perlolyrine	65.95	0.27	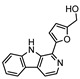
002151	senkyunone	47.66	0.24	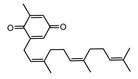
002157	wallichilide	42.31	0.71	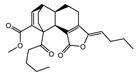
000359	sitosterol	36.91	0.75	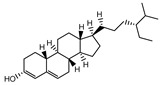
000433	Folic acid	68.96	0.71	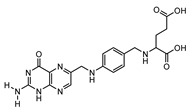
002122	(Z)-Ligustilide	53.72	0.07	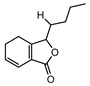
000360	Ferulic acid	39.56	0.06	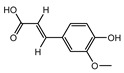
002208	Senkyunolide A	26.56	0.07	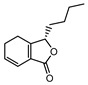
002202	tetramethylpyrazine	20.01	0.03	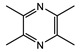
002127	Cnidilide	77.55	0.07	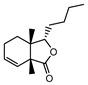
002111	Butylidene phthalide	42.44	0.07	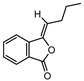
002143	senkyunolide I	46.8	0.08	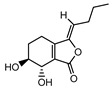
002200	Levistolide-A	9.96	0.82	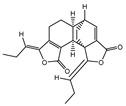
011770	senkyunolide J	42.34	0.1	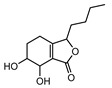
011771	senkyunolide L	10.68	0.09	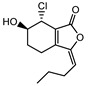
NA	3-butylphthalide	NA	NA	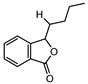

^a^ Oral bioavailability; ^b^ drug likeness.

**Table 2:** Results of docking scoring of active components of CX.

Target	Compound Name	Vina Core
4YLQ	Mandenol	−6.20
	Myricanone	−7.50
	Perlolyrine	−7.60
	Senkyunone	−7.20
	Wallichilide	−7.90
	Sitosterol	−7.40
	Folic Acid	−8.40
	(Z)-Ligustilide	−6.60
	Ferulic acid	−7.20
	Senkyunolide A	−6.60
	tetramethylpyrazinte	−5.70
	Cnidilide	−6.30
	Butylidene phthalide	−7.00
	Senkyunolide I	−7.10
	Levistolide A	−9.20
	Senkyunolide J	−6.30
	Senkyunolide L	−5.80
	3-butylphthalide	−6.50

## Data Availability

All data are contained in the article and the Supplementary Materials.

## Supplementary Materials

The following supporting information can be downloaded at: https://www.mdpi.com/article/10.3390/molecules28186702/s1, Table S1: The targets of candidate compounds related with thrombosis; Table S2: The degree values of core components; Table S3: The degree values of core targets; Table S4: GO analysis results; Table S5: KEGG analysis results; Table S6: The docking score of core compounds and core targets; Figure S1: The cytotoxicity of LIG.

## Sample Availability

Samples of the compounds are not available from the authors.
