# Supplementary material for: Exploring the Mechanism of Chuanxiong Rhizoma against Thrombosis Based on Network Pharmacology, Molecular Docking and Experimental Verification
Source: Molecules. 2023 Sep 19;28(18):6702. doi: 10.3390/molecules28186702 (PMC10535320; doi:10.3390/molecules28186702)
Supplement: Supplementary file 1 [file molecules-28-06702-s001.zip › molecules-2609485-supplementary.pdf]

Table S1 The targets of candidate compounds related with thrombosis

Table S2 The degree values of core components

Table S3 The degree values of core targets

Table S4 GO analysis results

Table S5 KEGG analysis results

Table S6 The docking score of core compounds and core targets

Figure S1 The cytotoxicity of LIG

Table S1 The targets of candidate compounds related with thrombosis

ID	Uniprot ID	Target name
1	P04035	HMGCR
2	P02768	ALB
3	P02652	APOA2
4	P23219	PTGS1
5	P35354	PTGS2
6	P29474	NOS3
7	P07550	ADRB2
8	Q03405	PLAU
9	P07900	HSP90AA1
10	P00915	CA1
11	P00533	EGFR
12	P49841	GSK3B
13	Q13547	HDAC1
14	P12259	F5
15	P05413	FABP3
16	P37231	PPARG
17	P28482	MAPK1
18	P11229	CHRM1
19	Q14432	PDE3A
20	P31645	SLC6A4
21	P21731	TBXA2R
22	P08254	MMP3
23	P53779	MAPK10
24	P45983	MAPK8
25	P00734	F2
26	P00751	CFB
27	P62937	PPIA
28	P15121	AKR1B1
29	P35228	NOS2
30	P08709	F7
31	P35968	KDR
32	Q16539	MAPK14
33	P08238	HSP90AB1
34	P20248	CCNA2
35	P09917	ALOX5
36	P04626	ERBB2
37	P34972	CNR2
38	P06241	FYN
39	P09211	GSTP1
40	P06276	BCHE
41	P16109	SELP
42	P53350	PLK1

43	P52732	KIF11
44	P00742	F10
45	P35398	RORA
46	P15692	VEGFA
47	Q16665	HIF1A
48	P08514	ITGA2B
49	P07477	PRSS1
50	Q99895	CTRC
51	P08581	MET
52	Q99572	P2RX7
53	P05362	ICAM1
54	P19320	VCAM1
55	P16581	SELE
56	P09601	HMOX1
57	P18031	PTPN1
58	P14555	PLA2G2A
59	P01375	TNF
60	P28223	HTR2A
61	O60674	JAK2
62	P05091	ALDH2
63	P08246	ELANE
64	P27986	PIK3R1
65	P05164	MPO

Table S2 The degree values of core components

ID	Compound name	Degree
1	Myricanone	25
2	(Z)-Ligustilide	17
3	Senkyunolide A	15
4	Cnidilide	10
5	Butylidene phthalide	10
6	Ferulic acid	9
7	tetramethylpyrazinte	8
8	3-butylphthalide	9

Table S3 The degree values of core targets

ID	Target name	Degree
1	ALB	51
2	TNF	40
3	VEGFA	39
4	PTGS2	37
5	MAPK1	36
6	MAPK8	33

Table S4 GO analysis results

Type	Name	No.	Target number
BP	response to nutrient levels	GO:0031667	19
	reactive oxygen species metabolic process	GO:0072593	18
	response to lipopolysaccharide	GO:0032496	18
	response to molecule of bacterial origin	GO:0002237	18
	blood coagulation	GO:0007596	15
	hemostasis	GO:0007599	15
	coagulation	GO:0050817	15
	regulation of reactive oxygen species metabolic process	GO:2000377	13
	response to nutrient	GO:0007584	13
	reactive oxygen species biosynthetic process	GO:1903409	11
CC	membrane raft	GO:0045121	13
	membrane microdomain	GO:0098857	13
	membrane region	GO:0098589	13
	vesicle lumen	GO:0031983	13
	secretory granule lumen	GO:0034774	12
	cytoplasmic vesicle lumen	GO:0060205	12
	caveola	GO:0005901	7
	plasma membrane raft	GO:0044853	7
	ficolin-1-rich granule lumen	GO:1904813	7
	blood microparticle	GO:0072562	7
MF	serine-type endopeptidase activity	GO:0004252	9
	serine-type peptidase activity	GO:0008236	9
	phosphatase binding	GO:0019902	9
	serine hydrolase activity	GO:0017171	9
	protein phosphatase binding	GO:0019903	8
	heme binding	GO:0020037	7
	tetrapyrrole binding	GO:0046906	7
	carboxylic acid binding	GO:0031406	7
	protein tyrosine kinase activity	GO:0004713	6
	MAP kinase activity	GO:0004707	4

Table S5 KEGG analysis results

No.	Pathway	Target number
hsa05418	Fluid shear stress and atherosclerosis	16
hsa04151	PI3K-Akt signaling pathway	14
hsa04933	AGE-RAGE signaling pathway in diabetic complications	12
hsa04510	Focal adhesion	12
hsa04668	TNF signaling pathway	11
hsa05167	Kaposi sarcoma-associated herpesvirus infection	11
hsa05205	Proteoglycans in cancer	11
hsa04657	IL-17 signaling pathway	10
hsa05215	Prostate cancer	10
hsa01521	EGFR tyrosine kinase inhibitor resistance	9
hsa04914	Progesterone-mediated oocyte maturation	9
hsa04659	Th17 cell differentiation	9
hsa04066	HIF-1 signaling pathway	9
hsa04611	Platelet activation	9
hsa04926	Relaxin signaling pathway	9
hsa04664	Fc epsilon RI signaling pathway	8
hsa04660	T cell receptor signaling pathway	8
hsa04370	VEGF signaling pathway	7
hsa04917	Prolactin signaling pathway	7
hsa05212	Pancreatic cancer	7

Table S6 The docking score of core compounds and core targets

Compound	Targets					
	MAPK1	TNF	MAPK8	VEGFA	PTGS2	ALB
3-Butylphthalide	-6.5	-5.4	-6.9	-4.5	-7.4	-6.5
Butylidene phthalide	-6.4	-5.7	-6.9	-4.5	-7.6	-8.1
Cnidilide	-6	-5.6	-6.9	-4.6	-7.5	-6.3
Ferulic acid	-5.7	-6	-6.1	-4.9	-7.3	-7.3
Myricanone	-7.9	-8.3	-8.1	-5	-7.6	-8
Senkyunolide A	-5.8	-5.6	-6.5	-4.3	-7.5	-6.4
Tetramethylpyrazine	-4.5	-4.7	-5.2	-3.2	-6.3	-5
Z-Ligustilide	-6.4	-5.5	-6.8	-4.4	-7.6	-8

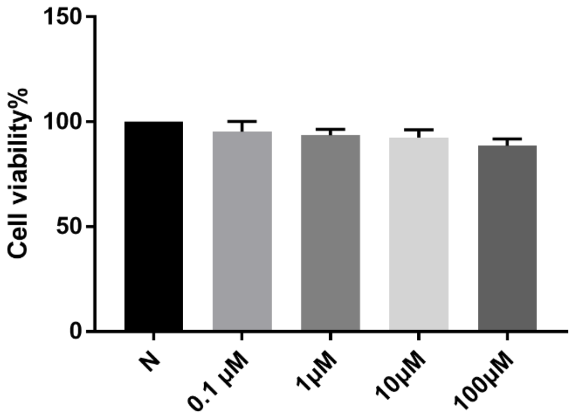

Figure S1 The cytotoxicity of LIG
